# The polymorphism of Insulin-like growth factor-I (IGF-I) is related to osteoporosis and bone mineral density in postmenopausal population

**DOI:** 10.12669/pjms.301.4264

**Published:** 2014

**Authors:** Li Yun-Kai, Wang Hui, Zhu Xin-wei, Guo Liang, Zuo Jin-liang

**Affiliations:** 1 Li Yun-Kai, The Fifth Surgical Department, The Fourth People’s Hospital, Jinan, 250013, China.; 2 Wang Hui, Department of Stomatology, Jinan Traditional Chinese Medicine Hospital,Jinan, 250012, China.; 3 Zhu Xin-wei, The Fifth Surgical Department, The Fourth People’s Hospital, Jinan, 250013, China.; 4 Guo Liang, Department of Radiotherapy, The Fourth People’s Hospital, Jinan, 250013, China.; 5 Zuo Jin-liang, The Fifth Surgical Department, The Fourth People’s Hospital, Jinan, 250013, China.

**Keywords:** Bone mineral density, Insulin-like growth factor-I, Osteoporosis, Polymorphism

## Abstract

***Objective:*** It has been shown that Insulin-like growth factor-1 (IGF-1) may be related with bone mineral density (BMD) or osteoporosis. But there are few evidences on the role of genetic variation of IGF-1 on the BMD or osteoporosis. We observed the relationship between polymorphisms of IGF-1(rs35767, rs2288377 and rs5742612) with osteoporosis and BMD in the postmenopausal female population in our study.

***Methods:*** A total of 216 postmenopausal women with a primary diagnosis of osteoporosis and 220 normal healthy women were included in the study. Genomic DNA of IGF-1 rs35767, rs2288377 and rs5742612 was extracted from the whole blood using QIAamp blood DNA mini kits (QIAGEN, Hilden, Germany) according to the methods recommended by the manufacturer.

***Results:*** We found that T allele of rs35767 had higher increased risk of osteoporosis (OR=1.34, 95%CI=1.0-1.81). Those carrying T allele of rs35767 had a significant lower BMD at L1–L4 vertebrae, femoral neck, total hip and trochanter when compared with those carrying C allele (*P* < 0.05). In addition, the BMD of L1–L4 vertebrae, femoral neck, total hip and trochanter decreased by 2.09%, 3.74%, 3.52% and 2.54% in women carrying T alleles compared with those carrying C alleles.

***Conclusion:*** Our study suggests that polymorphism in IGF-I rs35767 was significantly associated with BMD and osteoporosis in postmenopausal female population, and polymorphism of rs35767 could be a marker for lower BMD and risk of osteoporosis.

## INTRODUCTION

Osteoporosis is a skeletal disease characterized by compromised bone strength and an increased risk of fracture, especially for menopausal women.^1^ In the USA, osteoporosis occurs in 55% of the population aged ≥ 50 years.^2^ The incidence of osteoporosis is likely to rise as life expectancy increases. It is estimated, for example, that > 25% of the population in Canada will be aged ≥ 65 years by 2041.^3^ It is estimated that about 6.97% of the total 1.3 billion populations suffer from primary osteoporosis.^4^ Osteoporosis is skeletal disorder characterized by low bone mineral density (BMD) and loss of bone tissue that may lead to weak and fragile bones.

Although physical exercise and diet play important role in BMD, genetic factors strongly influence the BMD.^5^ It is reported that 60-80% of BMD may be determined by genetic factors.^6^ Previous studies reported that polymorphisms of vitamin D receptor, osteocalcin, collagen I, IL-6 and estrogen receptor may be associated with low BMD and osteoporosis.^7^

Insulin-like growth factor-1 (IGF-1) is a critical polypeptide for skeletal growth, which promotes bone cell growth, differentiation, cell cycle progression, and increases the activity of preexisting bone cells.^8^^-^^10^ It has been suggested that IGF-1 also acts as a mediator for various hormones which involved in the bone metabolism of postmenopausal women, including growth hormone (GH), estrogen and parathyroid hormone.^11^ IGF-1 could not only regulate bone size, shape and composition, but regulate individual’s ability to adapt its bone structure to mechanical loads.^12^ In addition, IGF-1 could promote osteoblast differentiation, mineralization and proliferation in vitro.^13^ However, previous studies also suggested no relationship between IGF-1 and BMD,^14^ and reported that the low levels of circulating IGF-1 had no actually relation with osteoporosis.^15^ Several SNPs of IGF-1 have been recognized to be involved in cancer, myopia, muscle function, BMD regulation and affects age at menarche.^16^^,^^17^ However, study investigated the links between polymorphisms of IGF-I and BMD or osteoporosis is lacking. Therefore, we conducted a study to observe the relationship between three common SNPs of IGF-1, rs35767, rs2288377 and rs5742612, and osteoporosis and BMD in the postmenopausal female population in our study.

## METHODS

***Subjects***: In this study, a total of 216 postmenopausal women with a primary diagnosis of osteoporosis were recruited from the fourth hospital of Jinan between May 2010 and November 2012. In the study women had to have a BMD T-score ≤ −2.5 at the femoral neck but no evidence of vertebral fractures, or a BMD T-score ≤ 1.5 with radiological diagnosis of two or more vertebral fractures. Patients with impairment of kidney and liver, severe endocrinological metabolic and those of who had received drugs known to alter bone metabolism were excluded from this study. At the same time, a total of 220 normal healthy non-pregnant women with reproductive age were included. Oral informed consent was obtained from all study participants. The participants in the study completed a questionnaire with information on medical and drug history, cigarette smoking, alcohol consumption and menopause status. 

Areal BMD (g/cm^2^) was measured by dual-energy X-ray absorptiometry (Hologic®, Waltham, MA, USA) at L1–L4 vertebrae, femoral neck, total hip and trochanter.

***Genotype analyses: ***5 ml venous blood was drawn from each cases and controls. The blood was kept in -20ºC, and EDTA with 1.5~2.2mg/ml was used for anticoagulant. Genomic DNA of IGF-1 rs35767, rs2288377 and rs5742612 was extracted from the whole blood using QIAamp blood DNA mini kits (QIAGEN, Hilden, Germany) according to the methods recommended by the manufacturer. Direct genome sequencing was used to separately analyze the SNP (rs35767). PCR and single base extension (SBE) primers were designed using Sequenom® Assay Design 3.1 software (Sequenom®), according to the manufacturer’s instructions.

Each PCR reaction mix comprised 50 ng genomic DNA, 200 μM dNTP, 2.5 U Taq DNA polymerase (Promega, Madison, WI, USA), and 200 μM primers, in a total volume of 20 µl. The cycling programme involved preliminary denaturation at 94°C for 2 min, followed by 35 cycles of denaturation at 94°C for 30 s, and annealing at 64°C for 30 s, with a final extension at 72°C for 10 min. For quality control, reproducibility was confirmed by repeating analysis of a randomly selection of 10% of the cases and controls.

***Statistical analysis: ***Continuous variables were presented as mean ± SD and analysed using independent sample t-test. Categorical variables were presented as n of subjects (%) and analysed using χ^2^-test. Deviations from Hardy-Weinberg equilibrium were evaluated by the χ^2^ test. Multiple linear regression analysis was employed to explore the association between genotype and osteoporosis, after adjusting for potentially risk variables (age, body mass index and smoking). χ^2^ test for trend was used to analyze the relationship between genotype and osteoporosis. All statistical analyses were performed using SPSS software (version 11.5; SPSS, Chicago, IL, USA). Statistical significance refers to *p* < 0.05.

## RESULTS

Of 216 patients and 220 controls, the mean age was 57.4±6.2 and 56.3±6.7 years (Table-I). Patients with osteoporosis were more likely to have higher age, higher weight, and lower BMD in L1–L4 vertebrae, femoral neck, total hip and trochanter (P<0.05).

**Table-I:** Basic characteristics of cases and controls

*Parameters*	*Cases (%)* *N=216*	*Controls (%)* *N=220*	*χ* ^2^ * or t value*	*P-value*
Age	57.4±6.2	56.3±6.7	1.77	0.04
Height(cm)	154.5±7.5	155.2±6.9	1.02	0.16
Weight(kg)	56.3±7.7	54.8±8.2	1.97	0.02
Smoking				
No	183(84.7)	197(89.5)		
Ever	33(15.3)	23(10.5)	2.26	0.13
BMD(g/cm^2^)				
L_1_–L_4_ vertebrae	0.91±0.091	0.93±0.110	1.86	0.03
Femoral neck	0.625±0.035	0.632±0.031	2.21	0.01
Total hip	0.64±0.049	0.65±0.047	2.24	0.01
Trochanter	0.58±0.041	0.59±0.049	3.48	<0.05

Data presented as mean ± SD or *n* (%) of patients.

^a^Parameters were compared between treatment groups by Student’s *t*-test for continuous variables or χ^2^-test for categorical variables.

The genotype and allele frequencies of IGF-1 rs35767, rs2288377 and rs5742612 were tabulated in Table-II. The Chi square test showed that the genotypic distributions of all three SNPs did not deviate from the Hardy-Weinberg equilibrium (*P* *>* 0.05). We found that T allele of rs35767 had higher increased risk of osteoporosis (OR=1.34, 95%CI=1.0-1.81). However, we did not find significantly effect of rs2288377 and rs5742612 on the risk of osteoporosis.

**Table-II:** The genotype and allele frequencies of IGF-1 and OR(95%CI) for osteoporosis

*IGF-I*		*Cases (%)* *N=216*	*Controls (%)* *N=220*	*OR(95% CI)* ^a^	*P-value*
rs35767	CC	95(44.0)	114(51.8)	1.0(Ref.)	-
-	CT	94(43.5)	89(40.5)	1.27(0.83-1.92)	0.24
	TT	27(12.5)	17(7.7)	1.91(0.93-3.96)	0.06
	C allele	284(65.7)	317(72.0)	1.0(Ref.)	-
	T allele	148(34.3)	123(28.0)	1.34(1.0-1.81)	0.04
rs2288377	AA	182(84.3)	189(85.9)	1.0(Ref.)	-
	AT	21(9.7)	21(9.5)	1.04(0.52-2.07)	0.91
	TT	13(6.0)	10(4.5)	1.35(0.53-3.53)	0.49
	A allele	385(89.1)	399(90.7)	1.0(Ref.)	-
	T allele	47(10.9)	41(9.3)	1.19(0.75-1.90)	0.44
rs5742612	CC	178(82.4)	183(83.2)	1.0(Ref.)	-
	CT	21(9.7)	21(9.5)	1.06(0.39-2.92)	0.90
	TT	17(7.9)	16(7.3)	1.09(0.50-2.39)	0.81
	C allele	377(87.3)	387(88.0)	1.0(Ref.)	-
	T allele	55(12.7)	53(12.0)	1.07(0.70-1.63)	0.76

^a^Adjusted for age, BMI and smoking

**Fig.1:**
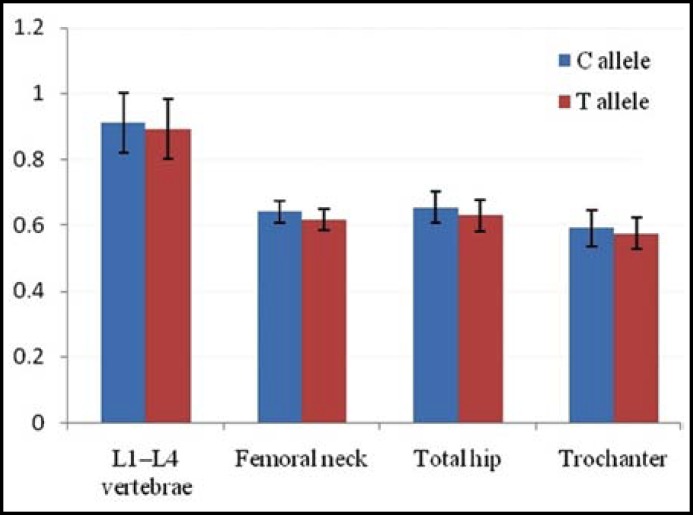
The BMD changes of rs35767 polymorphism at L1–L4 vertebrae, femoral neck, total hip and trochanter

We further evaluated the association between IGF-I rs35767 genotype and BMD values. We found T allele had a significant lower BMD at L1–L4 vertebrae, femoral neck, total hip and trochanter when compared with those carrying C allele (*P *< 0.05) (Table-III). In addition, the BMD of L1–L4 vertebrae, femoral neck, total hip and trochanter decreased by 2.09%, 3.74%, 3.52% and 2.54% in women carrying T alleles compared with those carrying C alleles (Fig.1).

**Table-III:** Bone mineral density of rs35767 polymorphism at the L1–L4 vertebrae, femoral neck, total hip and trochanter

*Location*	*Cases n = 216*		*χ* ^2^ * or t value*	*P-value*
	*C allele*	*T allele*		
L1–L4 vertebrae	0.92 ± 0.092	0.89 ± 0.090	3.24	< 0.05
Femoral neck	0.630 ± 0.034	0.617 ± 0.031	3.89	< 0.05
Total hip	0.645 ± 0.048	0.632 ± 0.047	4.64	< 0.05
Trochanter	0.589 ± 0.053	0.576 ± 0.049	4.05	< 0.05

## Discussion

Osteoporosis is a common disease affecting the majority of older women. It has been demonstrated that single nucleated polymorphism of some genes may associate with BMD and osteoporosis, such as Collagen I and VDR.^7^^,^^18^ In this study, we found that polymorphism of IGF-I rs35767 was associated with increased risk of osteoporosis, and we found T allele of rs35767 had a significant lower BMD at L1–L4 vertebrae, femoral neck, total hip and trochanter when compared with those carrying C allele.

It has been shown that genetic modulation is an important factor for bone phenotype parameters, such as bone mineral density, bone size and bone turnover.^6^ Genetic factors may explain 70% of the variance in bone phenotype.^6^ Life style, diet and hormonal factors interact with the genetic factor. Collagens I, estrogen receptor and VDR genotype have been shown to be related to bone density and to bone fracture.^7^

It is reported that IGF-I plays an important role in pathogenesis of osteoporosis.^19^ Low level of IGF-I may increase the risk of osteoporotic fractures in postmenopausal women and old men.^20^^,^^21^ Previous studies have revealed that IGF-I may be related with BMD and osteoporosis. Rosen et al (1998) showed that IGF-1 microsatellite (192bp) was related with decreased BMD at both the spine and hip, which indicated that there was a relationship between IGF-1polymorphism and BMD.^22^ But, Miyao et al. reported that there was no association between the microsatellite polymorphism of IGF-I gene and BMD in Japanese postmenopausal population.^23^ Therefore, the results between IGF-I polymorphism and BMD are conflicting. In our study, we found polymorphism of IGF-I rs35767 was associated with BMD and osteoporosis. Since these studies were conducted in different populations, direct comparisons between them are difficult to make. It can be presumed that the discrepancies may be due to differences in variant frequencies between races, and IGF-I polymorphism can play different role in the development of osteoporosis between populations.

Rs35767 site of IGF-1 has been shown to relate with various diseases, such as colorectal cancer, type 2 diabetes and muscle function.^16^ Previous studies demonstrated that IGF-1 polymorphism (rs35767) significantly associated with IGF-1 level.^19^ In our study, we found that IGF-I rs35767 T allele was associated with BMD and osteoporosis, which indicated that IGF-I polymorphism plays an important role in developing BMD and osteoporosis. Moreover, we also found that those women with T alleles would have significantly lower BMD compared with those with C alleles, and thus IGF-1 polymorphism may be a potential indicator for osteoporosis in postmenopausal women. Zhao et al revealed that polymorphism of IGF-1 rs6417 may affect age at menarche, but the polymorphism of IGF-1 rs35767 was not associated with age at menopause.^17^ Therefore, further large sample studies are greatly needed to clarify the association between IGF-1 rs35767 and BMD and osteoporosis.

***Limitation of our study: ***A limitation of our study is that it was conducted in a single hospital, and the participants may not have been representative of other populations. But the postmenopausal women and normal healthy non-pregnant women were randomly selected in our hospital, and the selection bias would be greatly reduced. Secondly, osteoporosis is influenced by multiple genetic and environmental factors, and thus other genetic and environmental factors should be considered in further studies.

## CONCLUSION

The results from the present research suggested that polymorphism in IGF-I rs35767 was significant associated with BMD and osteoporosis in postmenopausal female population, and polymorphism of IGF-I rs35767 could be a diagnostic marker for lower BMD and risk of osteoporosis. Further small sample size studies are greatly needed to confirm the association between IGF-I rs35767 polymorphism and risk of osteoporosis.

## Authors Contributions:

LYK and ZJL: Designed and performed the study, did statistical analysis & editing of manuscript.

WH, ZXW and GL: Did data collection and manuscript writing.
